# Absence of blood donors’ anti-SARS-CoV-2 antibodies in pre-storage leukoreduced red blood cell units indicates no role of passive immunity for blood recipients

**DOI:** 10.1007/s00277-023-05473-2

**Published:** 2023-09-27

**Authors:** Maddalena Casale, Maria Grazia Di Girolamo, Nicoletta Di Maio, Rita Tomeo, Martina Iengo, Saverio Scianguetta, Teresa Palma, Federica Porcelli, Saverio Misso, Silverio Perrotta

**Affiliations:** 1 Università degli Studi della Campania Luigi Vanvitelli, Naples, Italy; 2 Medicina Trasfusionale, ASL Caserta, Caserta, Italy

**Keywords:** SARS-CoV-2, Blood transfusion, Antibodies, Passive immunity, Packed red blood cells

## Abstract

Transfer of vaccine antibodies (Ab) from donors to recipients after transfusion of packed red blood cells (RBC) is supposed, thus affecting the recipients’ response to vaccinations. In this prospective study, SARS-CoV-2 IgG level in donors’ serum and RBC supernatant samples was assessed. Among 346 subjects, 280 were referred for hyperimmune plasma donation and 30 for whole blood donations. All units underwent pre-storage filtration, and residual plasma volume was 18±18 mL. The mean total IgG and IgM levels were 171.43 ± 48.79 and 11.43 ± 10.69 mg/dL respectively, with significant reduction after plasma depletion and filtration (IgG 5.86 ± 5.2 and IgM 1.43 ± 3.78, *p* < 0.05). Anti-COVID-19 Ab were identified in serum of 28/30 (93.5%) blood donors but were absent in all blood units. The mean value of anti-SARS-CoV-2 IgG level in donors’ serum samples and in RBC units was 8.80 S/C (range 0.01–23.4) and 0.11 (range 0.01–0.37) S/C, respectively (*p*<0.05). This study shows deplasmation and leukodepletion of RBC units ensured removal of IgG content and no red blood cell unit was reactive for anti-COVID-19 antibodies even from donors with high serum titre. These findings demonstrate that deplasmated and leukodepleted RBCs are not to be considered blood products containing substantial amounts of immune globulin, and differently from other blood derived-products containing Ab, transfusions with deplasmated and leukodepleted RBCs do not require delayed vaccinations and a revision of current recommendations is requested.

## Introduction

Blood transfusion is one of the most common hospital procedures, and in USA 11 million of red blood cell (RBC) units are collected every year [1].

Across the European Union (EU), 1400 blood establishments collect and process 20 million blood donations every year, enabling around 25 million transfusions to patients [2].

It has been reported that transfusion-dependent patients require 180 mL/kg/year starting from the first years of life [3], as high as 1–4 blood units every 4 weeks, or even more frequently in the case of acute events [4–6].

The COVID-19 pandemic and the subsequent vaccination campaign have determined an increase greater than 8-fold in the prevalence of seroreactive donations, and a continuous increase in this rate is expected [7]. These data confirm that a raising number of vaccinated and non-vaccinated blood donors have anti-COVID-19 Ab in their serum before donation.

One of the questions still to be answered is whether response to vaccinations in subjects who receive chronic blood transfusions can be altered by the passive transfer of donors’ Ab. The answer has important implications in clinical practice, in order to understand the impact of chronic transfusions on immunomodulation and response to vaccinations in transfusion-dependent patients [8–11]. Antibody-containing products can interfere with the immune response to vaccinations, and the duration of this interference is considered dose related, so as high is the value of Ab in the blood-derived product as longer is the interference with vaccine response. Packed RBCs are listed in the blood products containing substantial amounts of immune globulin, and an interval between transfusion and vaccinations is recommended, especially for live vaccines, but it is not clear the impact of Ab contained in blood derived products on other types of vaccines, leaving a considerable grey zone in this field [12].

Furthermore, transfer of Ab with blood products is a relevant topic in transfusion safety, since the most severe and life-threatening complications, such as transfusion-related acute lung injury (TRALI) cases, are associated with passive infusion of donor plasma containing antibodies [13–16]. Although packed RBCs are considered blood products with minimal residual plasma, there are multiple reports of TRALI after RBC transfusion which resulted from Ab in residual plasma [17–19] *.* A combination of a high-strength antibody and large residual plasma volume could explain severe or even fatal RBC-associated TRALI. However, no data are available on the residual Ab amount in leukoreduced RBC units, and reference institutions recommend a delay between RBC transfusion and administration on live attenuated vaccines up to 6 months, relying just on an assumed level of IgG in the RBC units and the IgG half-life [20, 21].

Zabeida et al. reported a significant immunity rate for measles, mumps, rubella vaccine in children on chronic transfusions, questioning the real need of immunization delay after RBC transfusion [22]. However, the unknown prevaccination immunity status in study population left open the question about the passive Ab transfer through RBC transfusions. So, the assessment of Ab and plasma levels in leukoreduced PRBC units is a relevant finding that may influence different aspects of transfusion medicine, such as immunomodulation and immune response in blood recipients.

The aim of this study was to examine the level of immunoglobulins, anti-SARS-CoV-2 Ab, and residual plasma in units of leukodepleted red blood cell (RBC) concentrates compared with the level of Ab in donor whole blood in order to evaluate the possible passive transfer of anti-SARS-CoV-2 Ab through transfusion of packed RBC units.

## Materials and methods

### Donor recruitment and screening

A public appeal was posted on the website of the Immuno-Transfusion Service of the local health authority in Caserta (Italy), to recruit potential donors after SARS-CoV-2 infection in January and February 2021. According to the Italian Ministerial Decree of 2 November 2015 and local approval (Ethics Committee Azienda Sanitaria Locale Caserta approval no. 735; 09/06/2020), donors were at least 18 years of age, met routine blood donor eligibility requirements, and had a history of COVID-19 with complete resolution of symptoms and two negative polymerase chain reaction (PCR) tests at least 14 days before screening. Informed consent was obtained from all participants. All procedures followed were in accordance with the ethical standards of the responsible committee on human experimentation (institutional and national) and with the Helsinki Declaration of 1975, as revised in 2008.

Data collected at the time of screening included age, sex, severity of SARS-CoV-2 infection, interval from COVID-19 symptom resolution to donation. Before donation, all donors were tested for SARS-CoV-2 IgG level and were referred for hyperimmune plasma donation or whole blood donation, according to the level of SARS-CoV-2 IgG. Subjects with higher titre SARS-CoV-2 IgG Ab (signal to cutoff values [S/C] ≥ 12) and SARS-CoV-2 neutralizing Ab titre ≥1:160 were referred for plasma donation; those with lower titre (S/C <12) for whole blood donation. Subjects with higher titre, following a first hyperimmune plasma donation, were deferred for 29 days and were allowed to donate whole blood after re-testing SARS-CoV-2 IgG Ab S/C value.

### SARS-CoV-2 IgG testing in donors’ plasma and RBC supernatant samples

SARS-CoV-2 IgG level in donors’ serum and RBC supernatant serum samples was assessed using the Ortho VITROS IgG assay, a chemiluminescent immunoassay (CLIA) that involves a two-stage reaction. Ortho VITROS is a highly specific assay for detection of anti-SARS-CoV-2 IgG, as all chemiluminescent techniques, and strongly correlates with ELISA assay and neutralizing antibodies (nAb) assay.

Initial anti-SARS-CoV-2 testing of donors and whole blood collection occurred for each donor the same day, at an interval not longer than 6 h.

The signal-to-cutoff values (S/C) increased as the amount of SARS-CoV-2 Ab present in the sample increased. The sample volume used per assay run was 80μL; the total sample volume required to run the assay was 115μL. S/C <1.0 indicated a sample non-reactive for anti-SARS-CoV-2 Ab; S/C ≥1.0 indicated a sample reactive for anti-SARS-CoV-2 Ab. The donors’ whole blood was collected in quadruple bags according to Good Practice Guidelines (GPGs) [12]. At least 2 h after collection, whole blood was centrifuged at 3.952g for 14 min at 22±2°C. After pre-storage filtration with Leucoflex LCRD2 filters (Macopharma Italy; Rho, Milan, Italy), RBC were centrifuged 3.952g for 10 min at 22±2°C (Heraeus Cryofuge 6000i, Thermo Scientific; USA, MA). The supernatant was then collected for anti-SARS-CoV-2 IgG assay. According to the Italian Ministerial Decree of 2 November 2015 and the European Directorate for the Quality of Medicines and Healthcare (EDQM) [12], all units underwent quality controls.

### IgG and IgM levels in RBC surnatant samples pre and post- deplasmation and filtration

To assess the capability of centrifugation, deplasmation, and leukodepletion to remove IgG and IgM, donors’ whole blood was obtained. The donors’ whole blood collected in quadruple bags was centrifuged at 3.952g for 14 min at 22±2°C. After automatic blood component separation (MacoPress SMART, Macopharma, France), RBC buffy coat depleted units were centrifuged at 3.952g for 10 min at 22±2 °C. The surnatant was removed, and an aliquot was used to measure IgG and IgM level. After deplasmation and leukodepletion by filtration of entire red blood unit in additive solution, RBC units were centrifuged at 3952g for 10 min at 22±2 °C, and the surnatant was collected to assess IgG and IgM level. The level of IgG and IgM in surnatant from RBC before and after deplasmation and leukodepletion were measured with immunoturbidimetric assay (Tina-quant® Immunoglobulin, Roche Diagnostics International Ltd, Rotkreuz, Switzerland).

Haemoglobin (Hb), haematocrit (HCT), haemolysis, and residual WBCs were determined before and after deplasmation and leukodepletion. Hb and HCT were determined by blood count (Sismex XN1000i by Dasit S.p.A., Cornaredo, MI, USA) and haemolysis by photometric method (HemoCue®Plasma/Low Hb System, Brønshøj, Denmark). The residual white blood cell count (r-WBC) was determined by fluorescence assay using ADAM r-WBC (Macopharma Italy; Rho, Milan, Italy). All methods were performed in accordance with the relevant guidelines and regulations.

### Estimates of residual plasma in PRBC units

RBC were manufactured by removal of plasma and storage in an additive solution (AS) and are not considered a high-plasma-volume component. The AS used was saline adenine glucose-mannitol (SAG-M), and the volume is fixed (either 100 or 110 mL). Therefore, the volume of residual plasma will depend on the centrifugation of the whole blood. The volume of residual plasma in RBCs does not have any clear specification or standard and is not subjected to any routine quality control. Each RBC unit was weighed, and the weight of the plastic container was tared. The volume of the unit was obtained by multiplying the tared weight in grams × 1065 to derive the volume of the RBC suspension in millilitres. A segment was taken from the container, after accurated stripping to uniform the sample, and the haematocrit (Hct) was measured using a cell counter (Sysmex XN-1000 by Dasit S.p.A., Cornaredo, MI, USA). With the Hct and total volume, the volume of RBC and supernatant was obtained. A quantity of 100 mL, relating to the additive solution, was subtracted to obtain the volume of residual plasma.

### Statistical analysis

Mann-Whitney-Wilcoxon test was used to compare differences in the values distribution between SARS-CoV-2 IgG level in donors’ serum and RBC supernatant samples and between the level of IgG and IgM in supernatant from pre- and post-filtration RBC [23]. Significance was determined at the *p*< 0.05 level.

## Results

A total of 346 subjects were screened for donation. After screening, 36 subjects were excluded because of low haemoglobin level (*n*=15), low pre-donation blood pressure (*n*=5), hypertension (*n*=4), drug therapy (*n*=5), sexual risk behaviours (*n*=2), personal decision (*n*=5 subjects), and 310 were referred to donation according to the Ab titre. SARS-CoV-2 IgG S/C ≥12 and SARS-CoV-2 neutralizing antibodies titre ≥1:160 were detected in 280 individuals, who performed hyperimmune plasma donation.

**Fig. 1:**
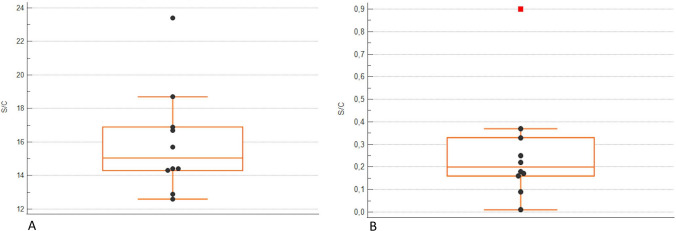
Anti-SARS-CoV-2 serology results in HIGH TITRE donors’ serum and correspondent red blood cell (RBC) units. Data are presented as box and whisker plots. **A**: Anti-SARS-CoV-2 IgG level in whole blood LOW titer donors; **B**: Anti-SARS-CoV-2 IgG level in leukoreduced RBC supernatant . IgG levels are reported in S/C, signal-to-cut-off. The central box covers the interquartile range with the median indicated by the line within the box. The whiskers extend either to the minimum and maximum values within 1.5 interquartile ranges of the quartiles. More extreme values are plotted individually

**Fig. 2:**
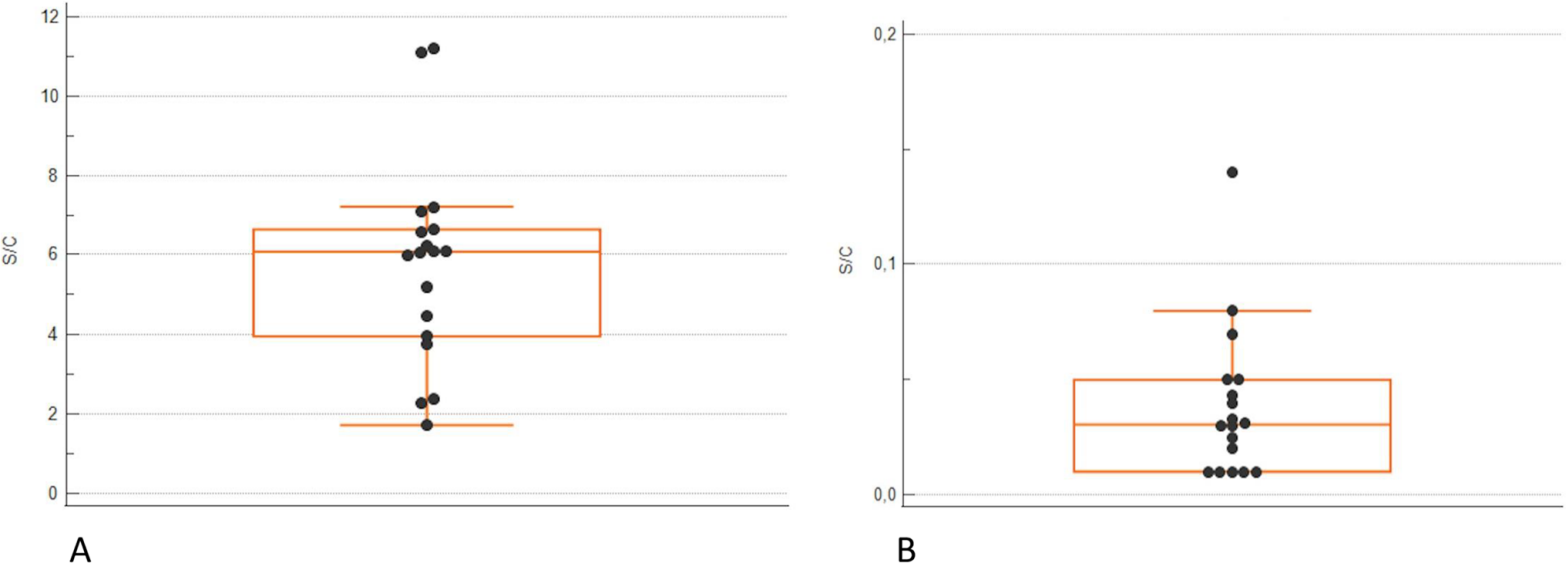
Anti-SARS-CoV-2 serology results in LOW TITRE donors’ serum and correspondent red blood cell (RBC) units. Data are presented as box and whisker plots. **A**: Anti-SARS-CoV-2 IgG level in whole blood LOW titer donors; **B**: Anti-SARS-CoV-2 IgG level in leukoreduced RBC supernatant . IgG levels are reported in S/C, signal-to-cut-off. The central box covers the interquartile range with the median indicated by the line within the box. The whiskers extend either to the minimum and maximum values within 1.5 interquartile ranges of the quartiles. More extreme values are plotted individually

Whole blood donations were performed by 2 individuals with non-reactive SARS-CoV-2 IgG samples [0.045 (0.01–0.08) S/C], 18 with reactive low IgG titre [6.08 (1.73–11.2) S/C] and 10 subjects with re-tested high titre SARS-CoV-2 IgG [15.05 (12.6–23.4) S/C], who were referred for whole blood donation at least 29 days after hyperimmune plasma donation (Table 1). All the correspondent leukoreduced PRBC units from the groups were non-reactive for SARS-CoV2 IgG, with residual level of 0.015 (0.01–0.02) in non-reactive group; 0.030 (0.01–0.14) in low titre group and 0.268 (0.01–0.37) in high titre group, respectively (Figs. 1 and 2).

**Table 1:** Characteristics of whole blood donors

	High titre	Low/non-reactive titre
Number	10	20
Titre S/C (range)	15.05 (12.6–23.4)	6.08 (1.73–11.2)
Male (%)	60	70
Female (%)	40	30
0 Rh +	2	5
A Rh +	3	6
A Rh −	1	5
B Rh +	1	1
B Rh −	2	1
AB Rh +	1	2
Asymptomatic Covid-19 infection	3	9
Symptomatic Covid-19 infection	6	5
Hospitalization for Covid-19 infection	1	6
Specific drug therapy	7	9
Interval from infection recovery and donation (days)	45	90

*S/C* signal to cutoff values

The mean value of total anti-SARS-CoV-2 IgG level in whole blood and in leukoreduced RBC units was 8.80 S/C (range 5.56–15) and 0.11 (range 0.01–0.11) S/C, respectively (*p*<0.05). The level of IgG, IgM, and other parameters were measured on surnatant from EC-BCD and EC-DPLD (Table 2).

**Table 2:** Change in different parameters before and after deplasmation and leukodepletion in packed red blood cell (RBC) units

Parameter	RBC buffy coat depleted units	RBC deplasmated and leudepleted units	*p* value
White blood cells (10^3^/μL)	9.92 ± 3.35	0.08±0.02	<0.05
Platelets (10^3^/μL)	238.33 ± 29.27	4.67 ± 1.03	<0.05
Haematocrit (%)	58.48 ± 1.24	71.20 ± 2.55	<0.05
Haemoglobin (g/dL)	22.92 ± 1.32	19.02 ± 0.81	<0.05
IgG (mg/dL)*	171.43 ± 48.79	5.86 ± 5.20	<0.05
IgM (mg/dL)*	11.43 ± 10.69	1.43 ± 3.78	<0.05

*Assessed on surnatant

The mean total volume of PRBC suspension was 291±26 mL, the mean PRBC volume was 173±19 mL, the mean Hct was 58.48±1.24%, the mean supernatant volume was 118±9 mL, the residual plasma was 18±18 mL, with a minimum plasma residual of 6 mL and maximum plasma residual of 34 mL.

All leukoreduced RBC units underwent quality controls according to the Italian Ministry of Health Decree of 2 November 2015 and national and international guidelines [7]. Mean r-WBC content was 0.08±0.02×10^3^ (Table 2).

## Discussion

The interaction between RBC transfusions and vaccinations is still controversial because no specific studies have addressed this tissue.

Reference institutions have suggested that recipients of chronic blood transfusions, such as those with TD thalassaemia, sickle cell disease, and other haematological diseases that require a regular transfusion regimen, can be partially protected by the transfer of Ab present in the donor’s serum. Although undemonstrated, there is a common belief that donor Ab can be transferred to the recipient through blood transfusion. In fact, as a precautionary measure, today it is still recommended to wait 3–6 months after transfusion of RBC concentrates before administering the measles, mumps, and rubella (MMR) vaccine because of possible interference from the passive immunity transferred by the blood units [24].

This interval cannot be adhered by TD patients, with the result that important vaccinations are missed, and patients and community can be placed at risk of preventable disease.

We have demonstrated that the quantity of total IgG present in leukoreduced RBC units was 10 times lower than that reported in previous studies, and that TD patients experienced a similar response to the MMR vaccine as non-transfused subjects [25], thus suggesting no interference between RBC transfusions and live vaccines.

In the present study, we aimed to examine the presence of Ig and anti-COVID-19 Ab in packed RBC units, in order to clarify if they can be considered blood products containing a substantial amount of immune globulins, as reported in the Recommendations and Reports of the Centers for Disease Control and Prevention [24] and can impact on response to vaccinations.

Approved RBC units for human use in most countries are prestorage leukoreduced either by filtration or buffy coat removal.

Plasmadepletion through fast centrifugation is the most relevant procedure to reduce Ab in packed RBCs, but to ensure the almost complete removal of plasma (99%) and its contents, the washing of RBCs can be required in specific conditions, with limited use of this product in transfusion medicine because of many drawbacks. However, our work demonstrated a significant reduction of Ab in packed RBCs after deplasmation.

Deplasmation of RBC units significantly reduces the number of residual plasma and IgG, and none of the collected units showed anti-COVID-19 Ab reactivity, even when blood donors had higher IgG titre.

Our study confirms no detrimental impact of chronic transfusions on immune response to vaccinations and adds new interesting findings, relevant in clinical practice for several reasons.

First, it excludes the presence of anti-COVID-19 Ab in the blood units transfused and refutes the possible transfer of protective Ab from donor to recipient. This means that patients and physicians must not have any false assurances about the role of passive immunity in TD subjects, who must adopt every available measure to protect themselves from vaccine-preventable diseases.

Secondly, it underlines the importance of guaranteeing full and free access to vaccination to subjects on chronic transfusion regimen since the absence of donors’ Ab in RBC units excludes any interference in the response to the vaccines by the recipient.

Our previous observations reported that TD patients present an excellent response to SARS-CoV2 mRNA vaccination, similar to healthy subjects, confirming no detrimental role of RBC transfusion [26]. However, antibody titres decrease more rapidly in TD patients than healthy subjects, and it is related to inflammatory and senescence-associated soluble mediators, which affect the durability of the humoral response to SARS-CoV2 mRNA vaccination [27]. These findings suggest a specific immune deficit in TD patients, and booster doses play an important role for maintaining antibody concentrations over time, as suggested in elderly subjects.

A recent review deeply analysed the immunogical impairment in thalassemia patients, and the complex link between chronic transfusions and the COVID-19 severity. TD patients present long-term iron overload complications, prolonged oxidative stress and immunosenescence but, surprisingly, develop less severe COVID19 infection compared to patients not on chronic transfusion regimen. No clear role of Hb level, serum ferritin, and iron overload was reported, and it was supposed a protective role of iron chelation therapy [28]. Our findings add another piece of the puzzle to elucidate the complex and multifarious interaction between chronic transfusions and COVID19 severity.

Besides the great improvements in the management of the TD disorders during the last decades [6, 29], infections remain a substantial cause of death in transfusion-dependent patients [30], and vaccinations represent the most formidable weapon against severe infective diseases. We demonstrated that leukoreduced RBC units, currently approved for human use, are blood derived-products with negligible amount of Ab; conversely, other antibody-containing blood products, such as intravenous immune globulin for replacement therapy in immune deficiencies or for treatment of immune thrombocytopenic purpura or Kawasaki disease, have a very high amount of Ab, with different impact on immune response to vaccinations. Intervals between administration of blood-derived products and vaccination must be recommended considering the real amount of antibodies in the blood products, and leukoreduced RBC units are to be considered blood products with minimal residual immune globulins.

In conclusion, although it is hypothesized that blood donors’ anti-COVID-19 antibodies (Ab) can be transferred to recipients of packed red blood cells (RBC), we demonstrated that no RBC unit was reactive for anti-COVID-19 Ab either from donors with low or high titre, and no passive immunity for recipients is plausible. Plasma depletion and filtration significantly remove Ab, and packed RBC units are not to be considered blood products with high amount of immune globulins. A revision of current recommendations about the interval between packed RBC transfusions and vaccinations is required.

## Data Availability

The datasets generated during and/or analysed during the current study are available from the corresponding author on reasonable request.
